# Redox-Driven Epigenetic Modifications in Sperm: Unraveling Paternal Influences on Embryo Development and Transgenerational Health

**DOI:** 10.3390/antiox14050570

**Published:** 2025-05-09

**Authors:** Aron Moazamian, Fabrice Saez, Joël R. Drevet, Robert John Aitken, Parviz Gharagozloo

**Affiliations:** 1 EVALSEM, GReD Institute, CRBC, Faculté de Médecine, Université Clermont Auvergne, 28 Place Henri Dunant, 6300 Clermont-Ferrand, France; fabrice.saez@uca.fr (F.S.); joel.drevet@uca.fr (J.R.D.); 2 CellOxess Biotechnology, Research & Development, Ewing, NJ 08638, USA; 3 Priority Research Centre for Reproductive Science, University of Newcastle, Newcastle 2308, Australia; john.aitken@newcastle.edu.au

**Keywords:** male infertility, oxidative stress, sperm DNA damage, epigenetics, 8-hydroxy-2′-deoxyguanosine (8-OHdG), embryo development, transgenerational health

## Abstract

Male-factor infertility accounts for nearly half of all infertility cases, and mounting evidence points to oxidative stress as a pivotal driver of sperm dysfunction, genetic instability, and epigenetic dysregulation. In particular, the oxidative DNA lesion 8-hydroxy-2′-deoxyguanosine (8-OHdG) has emerged as a central mediator at the interface of DNA damage and epigenetic regulation. We discuss how this lesion can disrupt key epigenetic mechanisms such as DNA methylation, histone modifications, and small non-coding RNAs, thereby influencing fertilization outcomes, embryo development, and offspring health. We propose that the interplay between oxidative DNA damage and epigenetic reprogramming is further exacerbated by aging in both the paternal and maternal germlines, creating a “perfect storm” that increases the risk of heritable (epi)mutations. The consequences of unresolved oxidative lesions can thus persist beyond fertilization, contributing to transgenerational health risks. Finally, we explore the promise and potential pitfalls of antioxidant therapy as a strategy to mitigate sperm oxidative damage. While antioxidant supplementation may hold significant therapeutic value for men with subfertility experiencing elevated oxidative stress, a careful, personalized approach is essential to avoid reductive stress and unintended epigenetic disruptions. Recognizing the dual role of oxidative stress in shaping both the genome and the epigenome underscores the need for integrating redox biology into reproductive medicine, with the aim of improving fertility treatments and safeguarding the health of future generations.

## 1. Introduction

Global fertility rates are projected to continue declining [1], with male factors recognized as a major contributor, accounting for nearly half of all infertility cases worldwide [2]. The steady decline in key parameters of male reproductive health—including sperm count [3], motility [4], and DNA integrity—raises serious concerns for fertility outcomes and transgenerational well-being [5]. Although numerous factors have been implicated in this decline, the precise molecular mechanisms remain only partially understood [6]. Notably, oxidative stress [7] has emerged as both a driver of sperm dysfunction and a critical determinant of genetic and epigenetic stability [8].

### 1.1. Redox-Driven Modifications in Spermatozoa

Spermatozoa exhibit a unique susceptibility to oxidative damage because of their structural and functional characteristics. Oxidative stress, defined by the imbalance between reactive oxygen species (ROS) and the body’s antioxidant defenses, generates oxidative DNA lesions such as 8-hydroxy-2′-deoxyguanosine (8-OHdG) [9]. Although more than 20 oxidative DNA base lesions have been identified [10], guanine ring is the most frequently oxidized base due to its low redox potential [11]. Hydroxyl radicals preferentially attack the C8 position of guanine’s imidazole ring, leading to the formation of 8-OHdG. Widely recognized as one of the most prevalent and mutagenic modifications, 8-OHdG has been implicated in de novo mutations [12] originating predominantly from the male germline [13]. Consequently, oxidative stress has been linked to male subfertility and infertility, increased sperm DNA damage, and an elevated miscarriage risk in women [14,15,16,17,18,19].

Beyond its mutagenic capability, 8-OHdG also plays a critical role in epigenetic regulation, illustrating its dual impact on both genomic and epigenomic stability [20]. In the male germline, oxidative stress can disrupt DNA methylation, histone modifications, and the integrity of small non-coding RNAs [21], as well as centromere [22] and telomere [23] integrity, ultimately affecting fertilization, embryo development, and offspring health [24,25]. These disruptions not only threaten genetic integrity but may also propagate epigenetic alterations [26] to subsequent generations.

Although reproductive biologists have devoted considerable effort to identifying genetic and epigenetic biomarkers predictive of male fertility and offspring health, this review focuses on the fundamental molecular processes linking oxidative stress, DNA damage, and epigenetic regulation to reproductive outcomes.

We examine the molecular interplay between oxidative stress, DNA damage, and epigenetic regulation within the context of male fertility. Central to this discussion is the pivotal role of 8-OHdG and its incomplete repair via the base excision repair (BER) pathway in driving genomic and epigenomic instability [27]. By integrating recent advances in redox biology, reproductive epigenetics, and DNA biochemistry, we propose a unifying framework to better understand male reproductive dysfunction and its potential impact on future generations.

### 1.2. A Roadmap to This Review

This narrative review begins with a concise overview of redox biology and epigenetic mechanisms, highlighting their individual roles in male fertility and early embryonic development (Section 2 and Section 3). It then explores how oxidative DNA lesions and their repair pathways mechanistically alter epigenetic regulation (Section 4). These discussions culminate in the proposal that oxidative stress significantly drives epigenetic changes in spermatozoa (Section 5). Subsequently, the review examines paternal epigenetic influences on embryo development and offspring health (Section 6) and concludes by discussing diagnostic and therapeutic approaches aimed at mitigating oxidative stress-related reproductive risks (Section 7).

## 2. Oxidative Stress and Male Fertility

An increasingly recognized concept known as ‘redox homeodynamics’ underscores the pivotal role of redox balance in shaping cellular responses to the broader exposome [28]. Oxidative stress emerges when reactive metabolic species (RMS) overwhelm endogenous antioxidant defenses (enzymes and small-molecule micronutrients), leading to cellular dysfunction [29] and molecular damage [30], affecting lipids, proteins, DNA, and RNA, and ultimately, if unchecked, causing cell death [31].

As germ cells mature, their DNA repair capacity declines, worsening further with paternal age [32]. Elevated oxidative stress impairs sperm motility and sperm–oocyte interactions, reducing fertilization rates [33]. Notably, significant sperm DNA damage occurs at oxidative stress levels lower than those required to disrupt fertilization itself [34]. These persistent genetic and epigenetic lesions may compromise offspring fertility and health. Thus, evaluating male reproductive competence should extend beyond fertilization alone, encompassing the potential to support successful pregnancy and healthy progeny.

The causes of oxidative stress in the male reproductive tract, particularly in sperm, are multifactorial. Endogenous contributors include ROS production by seminal leukocytes during immunological responses, sperm mitochondrial dysfunction, and enzymatic activity (e.g., NADPH oxidases, L-amino acid oxidase) [35]. Exogenous influences relate to modern lifestyle factors such as exposure to pollutants, heat, electromagnetic/radiation sources, and habits like smoking and alcohol consumption [36,37,38]. These stressors are further exacerbated by nutrient-deficient diets, a consequence of modern agricultural and food-processing practices [39].

### 2.1. A Unique Susceptibility

In spermatozoa, ROS have a dual role [40]: low-level production is essential for processes like sperm maturation, capacitation, tyrosine phosphorylation, and cAMP signaling, yet sperm are exceptionally vulnerable to oxidative stress due to their high polyunsaturated fatty acid membrane content (roughly 50% docosahexaenoic acid) [41,42], limited cytoplasmic antioxidant defenses [43], and truncated DNA repair mechanisms [27]. Consequently, these cells depend heavily on antioxidant protection within the seminal plasma and the reproductive tract [44]; any compromise in these defenses can lead to significant oxidative damage [45].

### 2.2. Far-Reaching Implications

When oxidative DNA lesions persist up to the point of conception and are not efficiently repaired by the oocyte—particularly if the oxidative burden is extensive and/or maternal repair capacity is also diminished—these 8-OHdG lesions may become fixed as de novo mutations/epimutations in the embryo [46]. Paternal age further amplifies this effect, as older fathers exhibit increased levels of oxidative stress and DNA damage, which correlate with elevated risks of neurodevelopmental disorders, psychiatric conditions, and other health vulnerabilities in offspring [47,48]. Recent findings also suggest that specific paternal genomic regions, enriched for genes of interest, are disproportionately susceptible to oxidative insults [49].

Clearly, oxidative damage to sperm extends beyond impairing fertilization, with significant implications for embryonic development and long-term offspring health. In the sections that follow, we will examine how DNA/RNA oxidative lesions disrupt critical epigenetic processes ranging from histone modifications and DNA methylation to the integrity of small non-coding RNAs, and how these disruptions can have profound, enduring consequences for future generations.

## 3. Epigenetics, Male Fertility, and Embryonic Development

Historically viewed as mere carriers of genetic information, spermatozoa are now recognized as key influencers of offspring health and disease risk through non-genetic mechanisms of inheritance [50,51]. Environmental factors and paternal age, among others, can impart heritable changes on sperm that extend well beyond DNA sequence alterations. Although epigenetic programs influence spermatogenesis and sperm maturation, multiple checkpoints tightly regulate progression from primordial germ cells to mature spermatozoa [52]. Consequently, this review does not focus on testicular epigenetic mechanisms but rather emphasizes post-testicular epigenetic modifications.

These epigenetic changes are mediated by classical processes—most notably histone modifications, DNA methylation, and small non-coding RNAs (sncRNAs)—and understanding how these processes regulate gene expression in ways that can persist through fertilization, escape reprogramming, and ultimately affect developmental and phenotypic outcomes in offspring is a rapidly advancing area of research [53].

### 3.1. Chromatin Modifications

During spermatogenesis, sperm DNA undergoes extensive reorganization, replacing most histones with protamines to compact the genome, protect it from oxidative insult, and maintain transcriptional silence [54,55,56]. A small proportion of histones remains, typically at genes essential for early embryonic development. These retained histones bear post-translational modifications, such as H3K4 methylation (active) and H3K27 methylation (repressive), that shape paternal epigenetic inheritance [57,58,59]. Protamines also undergo modifications (e.g., acetylation, phosphorylation) which influence sperm functionality and chromatin remodeling after fertilization [60]. Faulty protamine incorporation or abnormal P1/P2 ratios may impair pregnancy rates [61,62]; however, it remains unclear whether environmental influences on the paternal germline can alter protamine modifications sufficiently to impact embryonic development.

### 3.2. DNA Methylation

In sperm, DNA methylation involves the addition of methyl groups to cytosine bases, primarily at CpG dinucleotides, a process orchestrated by DNA methyltransferases (DNMTs) during spermatogenesis [63]. Ten-eleven translocation (TET) enzymes then refine these patterns by removing or modifying methylation marks [64]. Perturbations in sperm DNA methylation have been linked to infertility and reduced reproductive potential, underscoring the sensitivity of this epigenetic mechanism to environmental and physiological stressors [65,66,67,68]. Both histone/protamine modifications and DNA methylation patterns undergo two major waves of reprogramming, one during gametogenesis and another shortly after fertilization, to help safeguard the embryo from parental epigenetic disturbances [69,70]. Certain imprinted genomic regions, however, are exempt from these reprogramming events, limiting but not eliminating their transgenerational influence [71].

### 3.3. Small Non-Coding RNAs

Small non-coding RNAs, including microRNAs, piRNAs, and tRNA-derived fragments, represent another crucial layer of epigenetic regulation in sperm [72]. As regulators of chromatin architecture and gene expression, sncRNAs support fertilization, guide embryonic development, and establish broader developmental trajectories [73]. Unlike histone modifications and DNA methylation, sncRNAs are not extensively reprogrammed during gametogenesis or early embryogenesis, allowing them to directly convey environmental cues from the father to the embryo [74].

Growing evidence points to sncRNAs as potent mediators of transgenerational inheritance, linking paternal exposures, ranging from stress and diet to toxicants, with offspring phenotypes [75,76,77,78]. Through these molecular messengers, paternal experiences can influence the zygote’s genetic regulatory landscape and ultimately shape the health and development of subsequent generations.

The mechanisms underlying epigenetic alterations remain a critical area of investigation. Many factors implicated in epigenetic modifications—such as aging, environmental pollutants, and lifestyle choices—also drive oxidative stress, hinting at a shared redox-based mechanism. Over the past decade, this interplay between oxidative DNA damage, the cellular response, and epigenetic regulation has attracted significant attention. In the following section, we explore these specific processes, shedding light on the central role of redox stress in epigenetic regulation.

## 4. Crosstalk Between Oxidative Damage, Response, and Epigenetic Alterations

Beyond reproductive biology, there is broad scientific consensus that oxidative DNA lesions, particularly 8-OHdG, do more than simply mark DNA damage; they actively shape epigenetic regulation. This occurs via the direct actions of 8-OHdG, its processing by DNA repair enzymes, and their overlapping interactions with established epigenetic marks and processes [79].

### 4.1. 8-OHdG and Structural DNA Biochemistry

The BER pathway is critical for repairing oxidative DNA lesions. In somatic cells, 8-oxoguanine DNA glycosylase 1 (OGG1) initiates this pathway by excising 8-OHdG and leaving behind apurinic (AP) sites, which are then further processed by apurinic/apyrimidinic endonuclease 1 (APE1) and X-ray repair cross-complementing protein 1 (XRCC1) [80]. In addition to initiating repair, OGG1 and related BER enzymes help recruit transcriptional machinery, thereby influencing gene regulation [81,82].

When 8-OHdG in guanine-rich promoter regions is excised by OGG1, the resulting abasic site destabilizes the duplex, allowing the PQS to fold into a G-quadruplex (G4) and act as an epigenetic signal that activates transcription. Furthermore, when OGG1 engages 8-OHdG, it inserts catalytic residues, flips the lesion and its opposing cytosine out of the helix, and bends the DNA by ~70°. This structural remodeling creates a recognition platform that promotes the assembly of transcription-initiation complexes, thereby linking DNA repair to gene activation [82,83,84]. Genome-wide analyses also show that AP sites, OGG1, and APE1 are enriched within G4 sequences. Depleting APE1 abolishes cellular G4 formation, indicating that APE1 binding, and its acetylation-dependent residence time, promotes and stabilizes G4 folding. The resulting APE1–G4 complex then facilitates transcription-factor loading at promoters, revealing that oxidized-base repair not only safeguards genome integrity but also shapes higher-order DNA structures to modulate gene expression [85,86].

**Figure 1:**
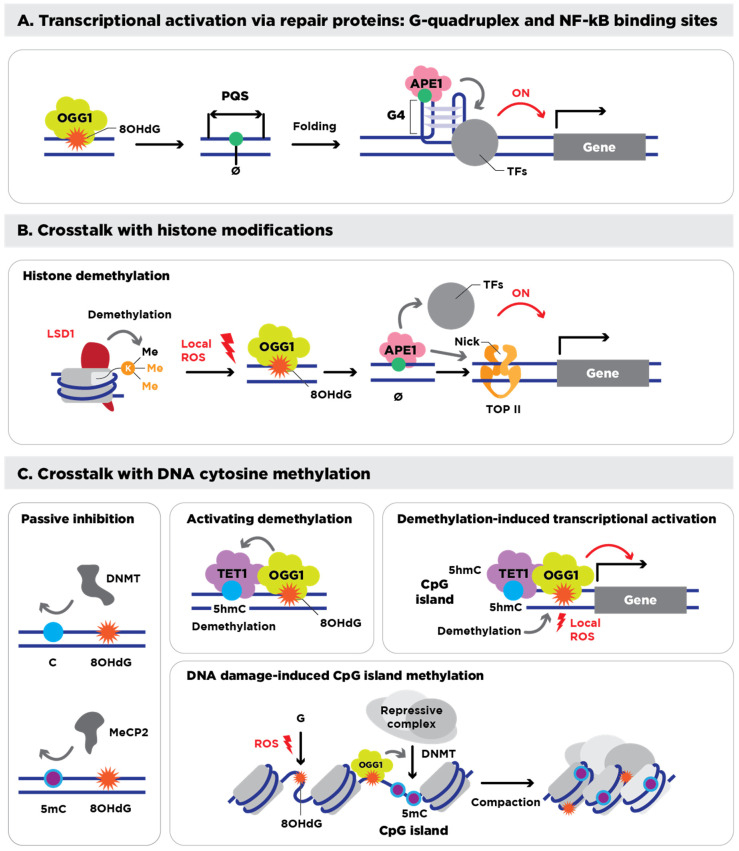
Epigenetic roles of 8-OHdG and its processing. (**A**) Transcriptional regulation mediated by 8-OHdG, its repair intermediate, AP site (Ø), and its repair proteins. 8-OHdG, bound by OGG1, and its intermediate AP site induce folding of quadruplex-forming sequences (PQSs) into a G4, which recruits various transcription factors (TFs) to transcriptionally activate downstream genes. (**B**) Interplay of 8-OHdG with epigenetic histone modifications. During the histone demethylation reaction, Lysine-specific demethylase 1 (LSD1) generates local ROS (H_2_O_2_) that lead to the formation of 8-OHdG and AP sites in the promoter, which are occupied by OGG1 and APE1. Then, APE1 recruits other TFs, and its nick formation associates with topoisomerase II (TOP II), eventually activating the transcription of downstream genes. (**C**) Interplay of 8-OHdG with DNA cytosine methylation (5mC). 8-OHdG near CpG islands inhibits the binding of DNMTs and methyl-CpG-binding protein 2 (MeCP2), thus passively interfering with 5mC (left panel). OGG1, which is associated with 8-OHdG, interacts with TET1, which oxidizes adjacent 5mC to 5-hydroxymethylcytosine (5hmC) for DNA demethylation (upper middle panel). During the DNA demethylation process of CpG islands, TET1 generates local ROS, which induce 8-OHdG associated with OGG1, thus activating the transcription of downstream genes (upper right panel). Oxidative DNA damage triggers the formation of the 8-OHdG and OGG1 complex, which recruits repressive complexes, including DNMT, and induces methylation of CpG islands, finally resulting in chromatin condensation and silencing of damaged DNA regions. Figure and legend adapted and reproduced from Hahm et al. [79].

Beyond transcription, persistent 8-OHdG lesions and AP sites also signal broader epigenetic changes [87]. OGG1 and APE1 collaborate with chromatin-remodeling complexes during repair [88,89,90,91], modifying genomic accessibility and facilitating other epigenetic processes, including methylation and demethylation. See Figure 1A,B for a summary of these processes. Differences in how the BER pathway functions in male versus female germ lines—as well as early embryos— reveal that while oxidative stress-driven epigenetic changes in sperm do not generally impair sperm functionality, they can dramatically alter gene expression patterns in the developing embryo and potentially compromise fertilization outcomes.

### 4.2. Redox-Mediated Methylation Processes

Oxidative stress also interferes with DNA methylation, a principal epigenetic mechanism [92]. DNMTs mediate the addition of methyl groups to cytosines in CpG islands, either preserving patterns during replication (Dnmt1) or establishing new marks (Dnmt3a and Dnmtb) [93]. Crosstalk between oxidative DNA damage and DNA methylation reveals a complex and multifaceted regulatory relationship involving 8-OHdG [94]. The presence of 8-OHdG can decrease DNMT binding affinity, indirectly inhibiting methylation, and in already-methylated CpG regions, can disrupt the binding of methyl-CpG binding proteins (MBPs) [95,96]. When 8-OHdG is converted to an AP site within potential G4 sequences, these structural changes can further modify local methylation dynamics [97].

Additionally, oxidative stress generates 8-OHdG lesions that recruit OGG1; OGG1, in turn, binds TET1 and delivers it to the damaged site, triggering local DNA demethylation. Cells lacking OGG1 resist this oxidative demethylation, whereas OGG1 over-expression enhances it, showing that BER machinery not only repairs 8-oxoG but also channels the demethylation signal to TET enzymes [98]. TET1 also initiates iterative oxidation reactions that demethylate 5-methylcytosine (5mC) into 5-hydroxymethylcytosine (5hmC) and subsequent oxidation products [99,100]. This process affects local DNA methylation patterns and modulates epigenetic reprogramming [101]. See Figure 1C for a summary of these processes. Altogether, these interconnected events showcase the duality of 8-OHdG as both a lesion in the genome and epigenetic modulator of methylation, blurring the lines between its genetic and epigenetic influences on embryo development and offspring health [102,103].

### 4.3. Implications for RNA Integrity

Because RNA is single-stranded and often lacks the protective proteins or robust repair mechanisms of DNA, it is inherently more vulnerable to oxidative damage. Guanine oxidation in RNA can affect both coding and non-coding transcripts and may be accompanied by strand breaks or crosslink formation—especially in small regulatory RNAs [104]. Site-specific oxidation of miRNAs or other sncRNAs can disrupt their structural integrity or binding specificities, ultimately dysregulating gene expression pathways linked to disease [105,106,107]. APE1—which is central to the BER pathway—also processes damaged RNA, including oxidized or apurinic forms, and can regulate oncogenic miRNAs [108]. Such oxidative modifications to RNA stem from a host of factors—exercise, drug abuse, toxins, inflammation, poor nutrition, and obesity—that are known to reshape the sncRNA landscape in sperm. These changes correlate with diverse offspring phenotypes, supporting a multi-layered network by which oxidative stress can propagate epigenetic changes across generations [109].

Taken together, these observations emphasize the pivotal role of 8-OHdG and DNA damage repair pathways in linking oxidative stress to epigenetic regulation. Through their influence on histone modifications, DNA methylation, and RNA integrity, oxidative lesions and their repair responses orchestrate dynamic changes that shape both immediate fertility outcomes and long-term heritable effects.

## 5. Oxidative Stress as the Key Driver of Epigenetic Change in Sperm

Accumulating evidence points to oxidative stress as a central player in genetic damage and altered epigenetic profiles in spermatozoa. Here, we argue that the distinct structural and functional characteristics of spermatozoa position oxidative stress as the primary mechanism shaping male subfertility and transgenerational health outcomes. This proposal rests on two foundational principles:

1. Inherent Susceptibility to Oxidative Damage: Spermatozoa exhibit an elevated vulnerability to oxidative insult due to their high polyunsaturated fatty acid content and limited antioxidant defenses. These features render their membranes, intracellular components (DNA, RNA, proteins), and epigenetic factors such as methylation and sncRNA signatures, exceptionally prone to oxidative damage or alteration [110]. In most cells, oxidative damage becomes problematic only when stress levels overwhelm robust repair pathways; spermatozoa, however, lack this, providing a fresh twist on the classic “two-hit” hypothesis.
2. Truncated BER Pathway: Unlike somatic cells, sperm rely on a partial BER process to address oxidative DNA lesions. Although they retain OGG1 for excising 8-OHdG, the lack of critical downstream enzymes such as APE1 and XRCC1 leads to the accumulation of apurinic sites and incomplete repair in the paternal genome [27]. Interestingly, these lesions may not prevent fertilization, yet they can still impact embryonic genetic and epigenetic programming, as their repair relies on the oocyte’s BER machinery.

The simultaneous presence of high oxidative damage and incomplete repair interrupts two key redox-mediated mechanisms observed in somatic cells: the direct effects of 8-OHdG and its downstream processing. As a result, oxidative stress can trigger the following epigenetic disruptions in sperm:

1. Impact of Increased Oxidative Damage:
  - Chromatin Architecture: Altered histone-to-protamine ratios can increase the susceptibility of interlinker DNA regions to oxidative assault [111], and escalating the consequence of these epigenetic modifications to further localized DNA oxidation, increased DNA fragmentation, lower fertilization rates, and poorer embryo quality [112,113].
  - G-Quadruplex Structures: The disruption of potential G4-forming sequences affects chromatin topology and the binding of epigenetic regulators [114,115]. Recent evidence indicates that G-quadruplex DNA adds another cis-regulatory layer to transcriptional control during embryonic development [116].
  - Methylation Dynamics: Perturbations in DNA methylation patterns and the balance between 5mC and 5hmC [117,118]. During early embryo development, the paternal genome undergoes rapid active DNA demethylation driven by TET enzymes, 5mC to 5hmC and then to 5-formylcytosine (5fC)/5-carboxylcytosine (5caC); Thymine DNA Glycosylase (TDG) excises these oxidized bases, and BER restores unmodified cytosine. Because this TET-TDG-BER machinery also repairs oxidative lesions, an excess of paternally inherited 5hmC or 8-OHdG could divert or saturate the pathway, reshaping the normal methylation-reprogramming wave and, in turn, altering embryonic gene regulation [119].
  - RNA Integrity: Up to 18,000 mRNAs and sncRNAs delivered by sperm may be compromised, influencing embryonic gene expression [120,121]. Recent studies confirm that specific sncRNA profiles correlate strongly with sperm quality and could serve as biomarkers to improve IVF success rates [122], alter the transcriptomic profiles of early embryos [123], and even predict contributions to regulation of gene expression in offspring development [124].
2. Consequences of APE1 Deficiency:
  - Chromatin Remodeling: Impaired recruitment of chromatin-modifying complexes hinders the proper establishment of histone marks and DNA methylation status [125,126,127,128]. APE1 spatiotemporal activity impacts both.
  - G4 Sequence Resolution: Prevalent oxidized bases, together with APE1, actively shape higher-order DNA structures that modulate transcription—an influence that extends beyond APE1′s traditional role in genome maintenance. Insufficient APE1 activity prevents the formation or resolution of G4 structures, further altering epigenetic regulation [23,85].
  - RNA Damage Processing: Emerging evidence shows that APE1, beyond its DNA repair duties, also participates in RNA metabolism: during genotoxic stress, APE1 associates with the DROSHA complex to influence miRNA processing and stability [129]. Deficient capacity to process oxidatively damaged RNA potentially alters transgenerational inheritance [130,131].

When these oxidative and epigenetic disruptions converge—particularly under conditions of advanced maternal age, where oocyte repair capacity is often diminished—the potential harm to embryonic development and offspring health is magnified. Collectively, the multifaceted role of 8-OHdG in both genetic and epigenetic processes underscore its significance as a key driver of male fertility issues and transgenerational health risks [132].

## 6. The “Perfect Storm” of Aging: Oxidative Damage, Impaired Repair, and Resulting (Epi)mutations

Spermatozoa, once they have matured, lose most of their capacity for DNA repair and possess minimal antioxidant defenses. As a result, genomic damage from a combination of endogenous, environmental, and lifestyle factors can accumulate over time with no inherent mechanism to rectify these lesions [27,133,134]. Consequently, the oocyte’s active DNA repair programs become essential for safeguarding the embryo from paternal mutagenesis [135,136]. The repair of the paternal genome requires the re-establishment of the nucleosomal chromatin architecture—replacing protamines with transition proteins and eventually histones [137]—thereby granting repair factors access to damaged DNA [138].

### 6.1. Oxidative Damage and Repair Post-Fertilization

For oxidative DNA lesions, repair in the zygote is initiated by 8-OHdG excision by OGG1 [139]. However, oocytes typically express OGG1 at relatively low levels, which limits this crucial initial step [139]. Intriguingly, fertilizing sperm deliver a significant complement of OGG1 to the zygote, thereby boosting its total repair capacity [27,138]. The subsequent steps—processing of apurinic sites—depend on APE1 and XRCC1, both abundant in oocytes but absent in sperm. Although this gametic collaboration theoretically curbs the risk of inheriting oxidative lesions, it also introduces a critical window where the same BER enzymes influence epigenetic processes. Paternal and maternal oxidative stress can thus have a profound impact on early embryonic development [140,141].

Nevertheless, an oocyte’s capacity to repair DNA diminishes with advanced maternal age [142,143], largely due to declining levels of key repair enzymes, including OGG1 [144]. The convergence of paternal and maternal aging thus creates a “perfect storm”—amplifying oxidative DNA damage in sperm while limiting the oocyte’s ability to repair it. This leads to higher risks of genomic instability in the embryo, as apurinic sites and 8-OHdG lesions may remain unresolved following fertilization [145,146,147]. Although the “post-meiotic collusion hypothesis” provides a compelling framework, direct experimental evidence detailing how oxidative stress in sperm affects DNA repair in the oocyte and early embryo remains limited, and the precise role of the oocyte’s DNA damage response fidelity has yet to be fully elucidated.

Although few clinical or in vivo studies have examined how advancing paternal and maternal age jointly influence heritable (epi)mutations, pregnancy data show a clear additive age effect: live-birth rates decline most when both partners are older [148]. Meta-analyses further indicate that advanced paternal age worsens ART outcomes when autologous oocytes are used, but not with donor oocytes—implicating co-existing maternal age as the main driver of these deleterious results [149].

### 6.2. The Paternal Origins of Health and Disease

Unresolved paternal genetic damage not only compromises embryonic developmental stability [150,151] but also has far-reaching implications for offspring health [152,153]. De novo mutations in paternal chromosome 15, a region notably susceptible to oxidative damage [49], are linked to numerous conditions, including developmental delays, autism spectrum disorders, epilepsy, schizophrenia, bipolar disorder, and multiple congenital anomalies [154,155,156,157,158,159,160,161,162,163,164,165]. The whole-genome sequencing of parent-offspring trios further reveals that children of older fathers exhibit higher frequencies of C>T transitions at CpG sites than those of younger fathers [166], reinforcing oxidative stress as a key driver of adverse transgenerational inheritance. Because the male germ line has a lower baseline mutation rate than somatic tissues, yielding only about two de novo mutations per year of paternal age, epigenetic disturbances largely explain the broader spectrum of outcomes triggered by oxidative damage in spermatozoa [167].

Beyond direct genomic instability, the interplay between advancing parental age, unrepaired oxidative damage, and decreased repair capacity disrupts the extensive epigenetic reprogramming critical for early embryogenesis [168]. During this stage, DNA methylation and histone modifications are restructured to establish a pluripotent state. The tight interconnection among oxidative stress, BER, and methylation/demethylation has been recognized as central to germline reprogramming events [169]. Specifically, the paternal genome becomes a primary target for TET enzymes—which facilitate active DNA demethylation via 5mC oxidation to 5hmC—but can be hindered by unresolved 8-OHdG lesions [170]. Similarly, methyltransferase machinery (e.g., DNMT1/2), dependent on chromodomain helicase DNA-binding protein 4 (CHD4) and OGG1 co-localization [98,171], may be diverted by ongoing oxidative repair processes. Recent experiments in bovine models also revealed that oxidative DNA lesions in the paternal genome impair active zygotic DNA demethylation through the preferential recruitment of XRCC1. This finding indicates that oxidative DNA damage repair is prioritized, potentially at the expense of concurrent epigenetic reprogramming processes [172]. Clearly, oxidative damage and its processing may contribute to aberrant epigenetic landscapes in the developing embryo [173].

A wide range of environmental and lifestyle factors known to induce oxidative stress—such as heat [78], poor diet [174], alcohol use [175], pollutants, endocrine-disrupting chemicals (e.g., phthalates) [176], and smoking [177]—are also implicated in altering sperm small non-coding RNA profiles. These changes have been linked to disruptions in embryonic development and DNA methylation as well as a host of offspring disorders, including metabolic, neurological, and behavioral abnormalities [178,179,180,181].

**Figure 2:**
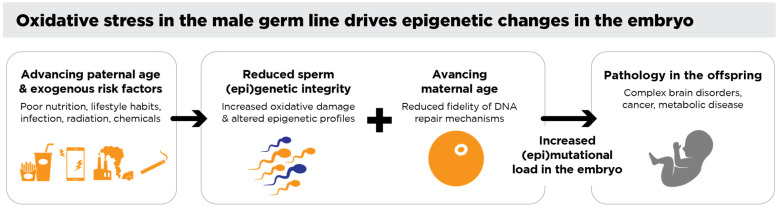
Oxidative stress in the male germ line drives epigenetic changes in the embryo. Environmental, lifestyle, and medical factors contribute to oxidative stress in the male germ line, leading to DNA damage and altered epigenetic profiles in spermatozoa. When a spermatozoon with significant oxidative DNA damage fertilizes an oocyte, the oocyte attempts to repair this damage during the critical period between fertilization and the initiation of the S-phase of the first mitotic division. Errors in this repair process can fix paternal oxidative DNA damage as mutations in the embryo and/or disrupt normal epigenetic reprogramming. Such damage may affect genomic regions, such as chromosome 15, associated with offspring pathologies including brain disorders, imprinting errors, and cancers—all of which are known to have a strong paternal component. Additionally, altered sperm-borne small non-coding RNAs (sncRNAs) have been shown to influence offspring health, further underscoring the transgenerational impact of oxidative stress in the male germ line.

Taken together, these genomic and epigenetic instabilities highlight the profound influence of preconception oxidative stress on offspring health (Figure 2), highlighting the urgent need for preventative and therapeutic strategies [182]. Addressing oxidative stress in both parents before conception may prove instrumental for improving reproductive outcomes and promoting long-term transgenerational well-being.

## 7. Navigating Antioxidant Therapy in Male Fertility: Harnessing Benefits While Mitigating Risks

Mounting evidence emphasizes the critical role of oxidative stress in undermining male fertility, compromising genomic and epigenetic integrity, and potentially impairing embryonic development. Against this backdrop, targeted antioxidant therapy has emerged as a highly promising intervention capable of neutralizing RMS and reducing oxidative DNA lesions that accumulate in sperm [183]. By proactively mitigating oxidative damage, antioxidant formulations may help safeguard the paternal genome, preserve key epigenetic marks, and optimize reproductive outcomes in both natural conception and ART.

While conceptually promising, robust evidence from human clinical trials demonstrating its efficacy in improving fertility outcomes remains limited [184,185]. However, all of those trials share a critical limitation: subjects were enrolled without first confirming seminal oxidative stress. Without that screening, men who did not need antioxidants were mixed with those who did, diluting any therapeutic effect and in fact potentially causing adverse effects [186]. Indeed, both clinical observations and in vitro studies show that antioxidant supplementation in males without demonstrable oxidative stress can be counter-productive, lowering sperm quality and potentially compromising overall fertility [187,188,189,190,191,192].

Critically, the latest studies support a precision medicine approach rather than a one-size-fits-all supplementation regimen. Research by Moazamian et al. highlights the dual role of antioxidants: while high-dose micronutrients significantly enhanced sperm DNA integrity in oxidatively stressed mice, they paradoxically caused redox imbalances, oxidative DNA damage, and DNA fragmentation in healthy controls with normal redox status [193]. Similarly, work by Hug et al. indicates that antioxidant supplementation can normalize broad epigenetic patterns in men under oxidative stress yet alter typical epigenetic programming in those without elevated stress levels [102]. Such findings underline the importance of oxidative stress assessments to tailor antioxidant therapy—ensuring that the supplementation matches an individual’s redox requirements without inducing reductive stress.

In practical terms, this means adopting the following guidelines for effective, safe, and evidence-based antioxidant therapy in male fertility care:

- Screening and Diagnostics: Employ reliable biomarkers (e.g., ROS levels, 8-OHdG measurements, antioxidant status) to either measure DNA damage directly [12] or the oxidative stress level in the semen [194] to identify individuals most likely to benefit from antioxidant interventions.
- Targeted Formulations: Employ carefully formulated [163], evidence-based antioxidant supplements designed to counter exposures known to induce oxidative stress [195,196].
- Controlled Dosage: Calibrate dosing to mitigate oxidative damage without triggering reductive stress [193] or disturbing normal epigenetic landscapes [102].

When implemented correctly, antioxidant therapy holds immense potential for bridging a critical gap in current fertility treatments, enhancing sperm quality, and ensuring the genetic and epigenetic health of future generations [186]. As scientific understanding of the redox–epigenetic axis continues to evolve, innovators in the field stand poised to refine these targeted antioxidant strategies and transform them into a cornerstone of modern male fertility management.

## 8. Conclusions

Oxidative stress represents a critical yet underappreciated threat to male reproductive health, exerting profound effects on both the genome and epigenome. At the heart of this process is 8-OHdG, which functions both as a marker of oxidative damage and an active epigenetic regulator. This dual capacity underscores its importance in shaping heritable traits, as 8-OHdG-driven alterations can propagate through multiple generations.

The heightened vulnerability of spermatozoa to oxidative stress, combined with its truncated base excision repair pathway, magnifies the impact of 8-OHdG-induced damage. Disruptions to DNA methylation, post-translational modifications of histones and protamines, and the integrity of small non-coding RNAs all converge to undermine classical epigenetic regulation and reprogramming, ultimately affecting embryonic development and long-term offspring health.

Clinically, these insights make a compelling case for embracing redox biology within reproductive medicine. By systematically identifying and mitigating oxidative stress, via precision-based antioxidant therapy and other targeted interventions, healthcare professionals can substantially improve fertility outcomes and safeguard the well-being of future generations. This holistic integration of oxidative stress management into standard clinical practice stands to reshape the landscape of fertility treatments and holds transformative potential for advancing transgenerational health.

## Data Availability

No new data were created or analyzed in this study. Data sharing is not applicable to this article.

## Abbreviations

The following abbreviations are used in this manuscript:

8-OHdG	8-hydroxy-2′-deoxyguanosine
RMS	Reactive metabolic species
sncRNAs	Small non-coding RNAs
DNMTs	DNA methyltransferases
TET	Ten-eleven translocation
OGG1	8-oxoguanine DNA glycosylase 1
APE1	Apurinic/apyrimidinic endonuclease 1
XRCC1	X-ray repair cross-complementing protein 1
MBPs	Methyl-CpG binding proteins
5mC	5-methylcytosine
5hmC	5-hydroxymethylcytosine
5fC	5-formylcytosine
5caC	5-carboxylcytosine
TDG	Thymine DNA Glycosylase
CHD4	Chromodomain helicase DNA-binding protein 4
